# Nonsurgical root canal treatment of an Oehlers type IIIa maxillary central incisor with dens invaginatus: a case report

**DOI:** 10.3389/fdmed.2024.1458215

**Published:** 2024-10-31

**Authors:** Ayumi Inoue, Yuzo Kawanishi, Motoki Okamoto, Mikako Hayashi

**Affiliations:** ^1^ Department of Restorative Dentistry and Endodontology, Osaka University Graduate School of Dentistry, Osaka, Japan; ^2^ Department of Oral Science and Translational Research, College of Dental Medicine, Nova Southeastern University, Fort Lauderdale, FL, United States

**Keywords:** cone-beam computed tomography, dens invaginatus, dental operating microscope, single-cone hydraulic condensation, bio-ceramic root canal sealer

## Abstract

Dens invaginatus has a complex anatomy, making endodontic treatment challenging. We report the case of a 11-year-old girl who presented with radiolucency at the apex of the maxillary central incisor; she was diagnosed with symptomatic apical periodontitis. Cone-beam computed tomography (CBCT) revealed dens invaginatus. The invagination and root canal were observed using a dental operating microscope, and nonsurgical root canal treatment was performed. The invagination was connected to the periodontal tissue, and the tooth was classified as Oehlers type IIIa. Root canal preparations were performed using NiTi files and an ultrasonic device. A calcium hydroxide paste was used as an intracanal dressing. The root canal was filled using a single-cone hydraulic condensation technique with a highly fluid calcium silicate-based sealer. At the 2-year follow-up, no clinical symptoms were observed, and CBCT images revealed no radiographic lesions. Nonsurgical endodontic treatment using CBCT imaging, dental microscope, effective cleaning systems, and a highly fluid sealer facilitated the successful treatment of apical periodontitis attributed to dens invaginatus. The single-cone hydraulic condensation technique using a bio-ceramic sealer is considered effective even in cases with a complex morphology and open apex.

## 1 Introduction

Dens invaginatus presents as an abnormal formation of the enamel organ in the dental papilla during odontogenesis (1). While several mechanisms, such as infection, trauma, and growth pressure, have been proposed concerning the development of dens invaginatus, the definitive cause remains unclear (2). One study reported that the incidence of dens invaginatus ranged from 1%–10% (3). Furthermore, the usual site is the maxillary lateral incisor, with an incidence of 2.7%–9.66% (4), whereas other tooth types have an incidence of 0.01%–0.3% (5, 6).

Many patients with dens invagination are asymptomatic, and the condition is often discovered incidentally on radiographic images (3). Moreover, its complex morphology often makes the removal of the source of infection during nonsurgical root canal treatment challenging. The depth of the invagination also affects the degree of treatment difficulty. Most previous case reports concerning nonsurgical root canal treatment of dens invaginatus have focused on maxillary lateral incisors (7, 8), with few reports involving other tooth types (9).

In this case report, we report the successful nonsurgical root canal treatment of a rare case of dens invagination with an Oehlers classification of type IIIa, involving a maxillary central incisor with chronic apical periodontitis, which was successfully treated.

## 2 Case description

**Figure 1:**
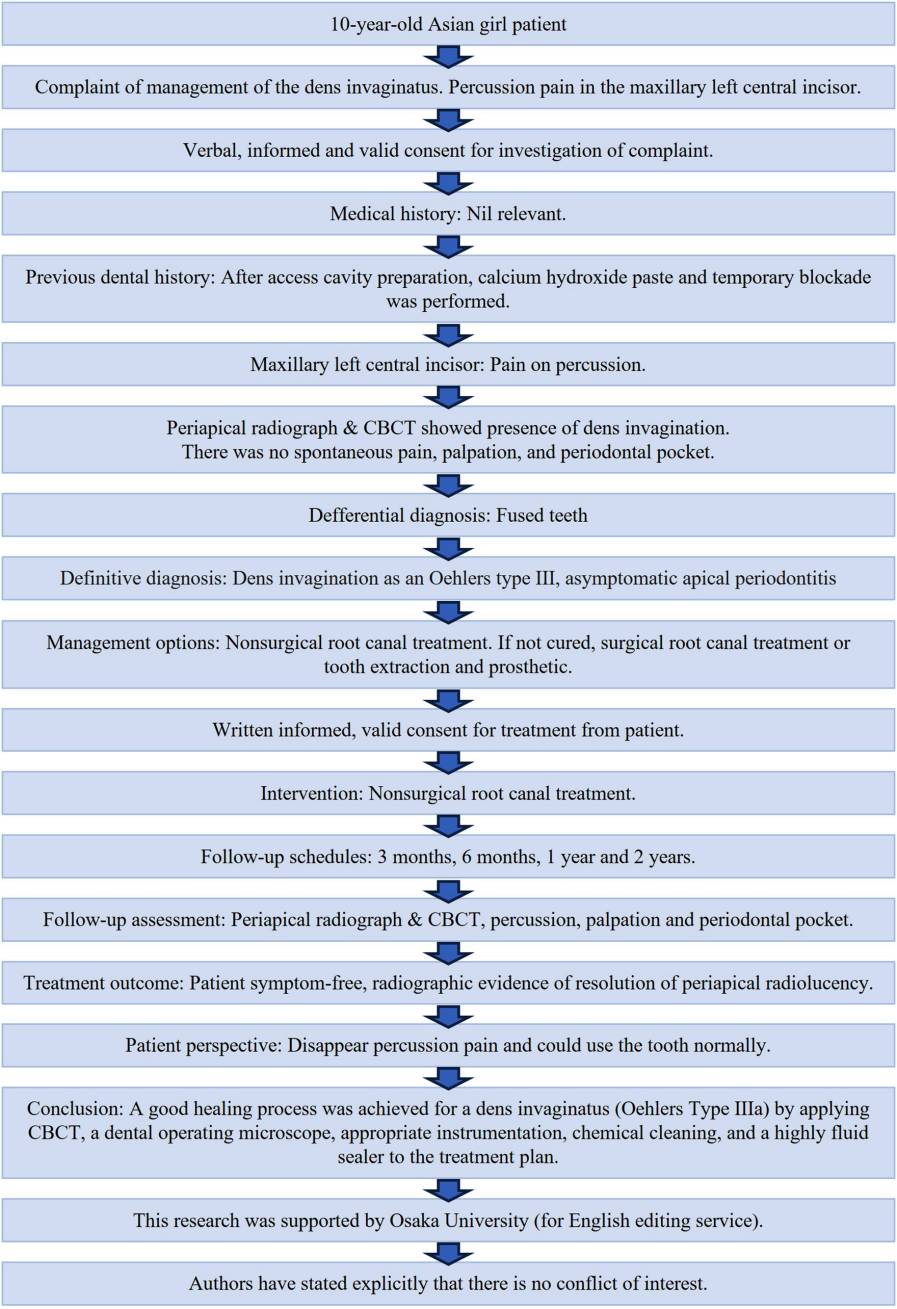
PRICE 2020 flowchart.

This case report is described in accordance with the Preferred Reporting Items for Case Reports in Endodontics (PRICE) 2020 guidelines (10). The PRICE 2020 flowchart is presented in Figure 1. The patient and her parents provided informed consent for the publication of this report.

**Figure 2:**
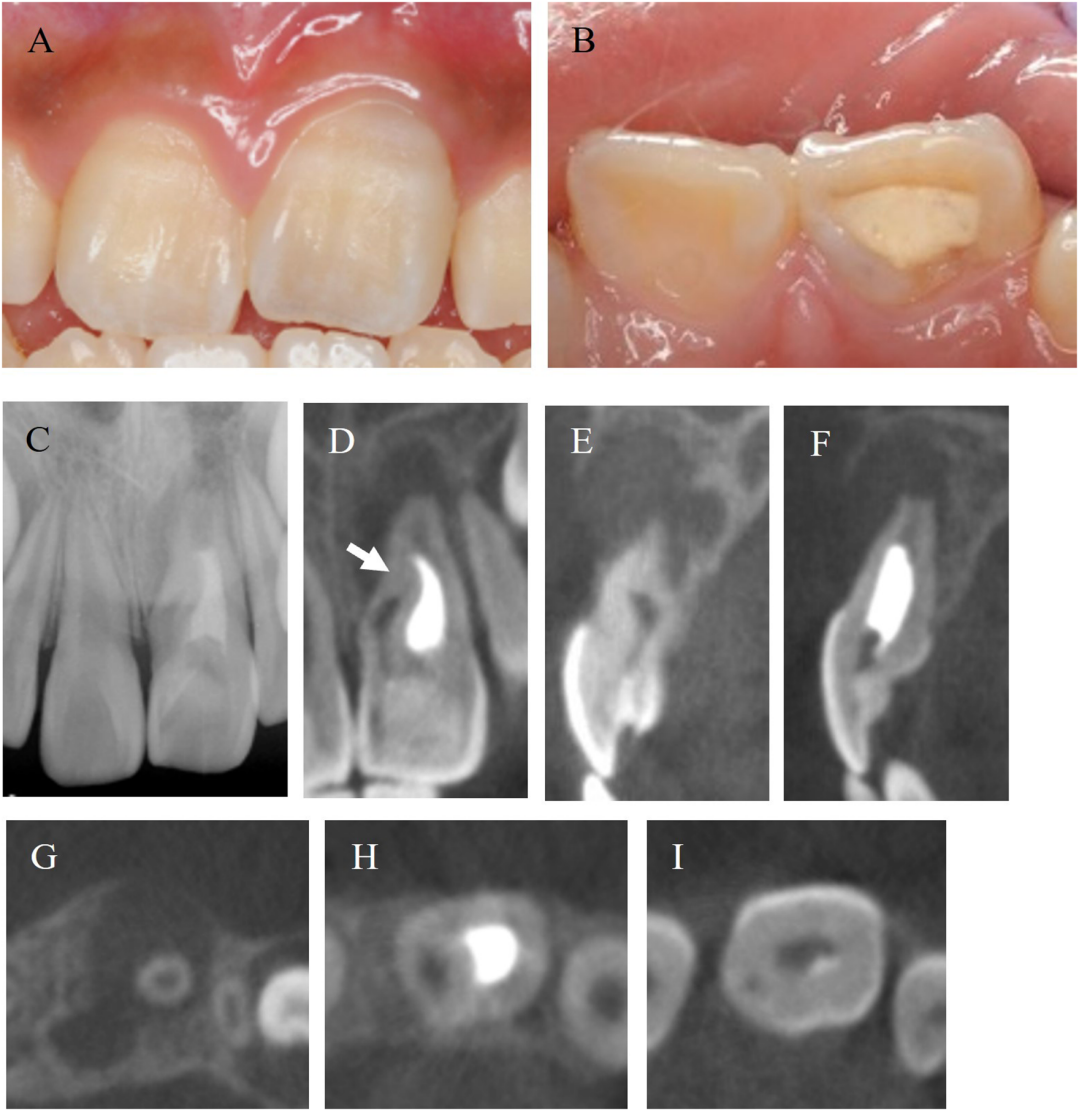
Intraoral images and preoperative radiographic images. **(A,B)** Labial and occlusal views of the maxillary central incisor of 11-year-old Asian girl. **(C)** Periapical radiograph of before treatment. **(D)** CBCT image of coronal cross section. Radiolucency surrounds the root apex. The invagination penetrated into the middle of the root (white arrow). **(E,F)** CBCT images of sagittal section from mesial to distal. Buccal cortical bone disappeared. **(G–I)** CBCT images of axial cross section from apical to coronal.

A 11-year-old Asian girl, with no relevant medical or family or psycho-social history, presented at a private dental clinic with spontaneous pain in her left maxillary central incisor (Figure 2A). She was diagnosed with symptomatic apical periodontitis and nonsurgical root canal treatment was initiated. However, owing to the challenges in treating the dens invaginatus, she was then referred to our university hospital. The patient had no history of trauma or orthodontic treatment for the tooth with invaginatus. The upper anterior teeth were crowded, and the patient had never undergone routine dental treatment; thus, the oral hygiene was poor.

## 3 Diagnostic assessment and treatment

On the first visit to our university hospital, the patient reported no spontaneous tooth pain or tenderness on palpation, except for percussion pain. Periodontal probing depths and tooth mobility were within normal limits. Misaligned teeth and poor oral hygiene were observed. The left maxillary central incisor had been temporarily sealed at the private dental clinic (Figure 2B). A periapical radiograph showed radiolucency at the root apex and an enlarged root apex in the maxillary left central incisor, and intracanal medication was noted in the root canal (Figure 2C). Cone-beam computed tomography (CBCT) images revealed that the invagination extended to more than one-third of the root in the medial region (Figure 2D). The radiolucency had a maximum diameter of 11.5 mm, and the buccal cortical bone was resorbed (Figures 2E–I). CBCT images (AlphardVEGA; ASAHIROENTGEN IND. CO., LTD., Kyoto, Japan) were obtained with a slice width of 0.1 mm.

Based on the above assessment, the patient was diagnosed with symptomatic apical periodontitis and dens invaginatus in the maxillary left central incisor. The treatment plan was discussed with both the patient and her parents, who were informed of the nonsurgical root canal treatment for both the root canal and invagination. If non-surgical endodontic treatment was ineffective, surgical endodontic treatment was considered. Oehlers type III has a complex root morphology and connection with the periodontal tissue; thus, there is a possibility of recurrence even after apicoectomy or intentional replantation. If these treatments did not improve the clinical symptoms, we planned to extract the teeth and provide prosthetic treatment. The proposed prosthetic treatment included the fabrication of a unilateral wing brace or partial dentures considering the patient's age; we obtained informed consent from the patient and their family. The treatment timeline is shown in Table 1.

**Table 1:** Timeline symptoms and treatment of maxillary central incisor.

	Clinical main symptoms	Clinical treatment
1-week previously (At private dental clinic)	Spontaneous tooth pain	Pulptomy
		Referred to a university hospital for dens invaginatus
Root canal treatment (At dental hospital)	Tenderness to the percussion	Root canal treatment
	No spontaneous tooth pain	
	No periodontal problems	
Root canal filling (At dental hospital)	No tenderness to percussion and palpation	Root canal treatment (Root canal filling)
	No periodontal problems	
Follow-up 3 months after root canal filling (At dental hospital)	No clinical symptom	Resin composite restoration for access cavity
Follow-up 6 months after root canal filling (At dental hospital)	No clinical symptom	
Follow-up 1 year after root canal filling (At dental hospital)	No clinical symptom	
Follow-up 2 year after root canal filling (at dental hospital)	No clinical symptom	

After local anesthesia (2% lidocaine with adrenaline 1:80,000; Dentsply Sirona, Chuo, Japan) and rubber dam isolation, the infected dentin was removed under a dental operating microscope (OPMI pico MORA/S100; Carl Zeiss, Land Baden-Württemberg, Germany).

**Figure 3:**
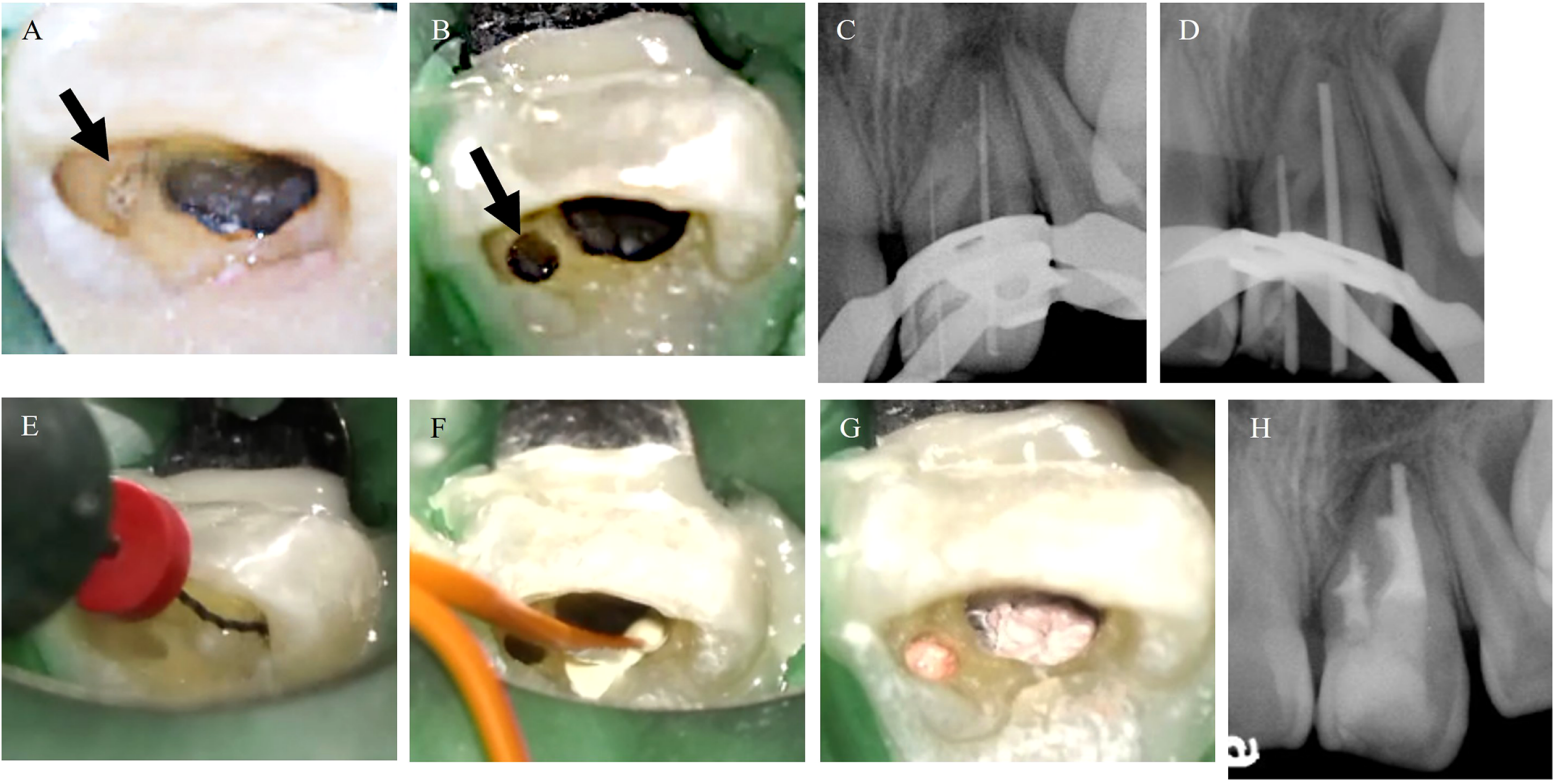
Photograph from video of dental operating microscope and periapical radiograph images during the treatments. **(A)** Before removing infected dentin, the invagination was observed (black arrow). **(B)** Orifice of the invagination (black arrow). **(C)** Periapical radiograph with initial file. **(D)** Periapical radiograph with master cones. **(E)** Root canal irrigation using XP-endo Finisher before root canal filling using Sodium hypochlorite solution and EDTA solution. **(F)** Root canal filling using Bio-C ® Sealer. **(G,H)** Photograph and periapical radiograph after root canal filling.

A depression was observed on the mesial side of the crown (Figure 3A); following insertion of a #8K file (Mani, Utsunomiya, Japan), this depression was determined to be the invagination. Access cavity preparation was performed in the root canals and invagination (HyFlex™ EDM Orifice Opener; Coltene, Sankt Gallen, Switzerland) (Figure 3B). The root canals were irrigated with 2.5% sodium hypochlorite (NaOCl) (Neo Cleaner; Neo Dental Chemical Products Co., Ltd., Shibuya, Japan) and 3% ethylenediaminetetraacetic acid (EDTA) (Smear Clean; Nishika, Shimonoseki, Japan). The root canals were dried using sterile paper points (Absorbent Paper Points; Zipperer, Hamburg, Germany). A calcium hydroxide pastes (Calcipex Plane II; Nishika) was then applied, and a temporary blockade (Caviton; G.C., Bunkyo, Japan) was performed.

At the second visit (5 months after the first visit), the patient reported that the percussion pain had disappeared. After local anesthesia and rubber dam isolation, the working length was determined using an apex locator (RootZX2; Morita, Suita, Japan). The invagination was probed using a file and an apex locator, which showed patency lateral to the periodontal tissue, indicating that the affected tooth was Oehlers type III. A periapical radiograph with initial files (Figure 3C) confirmed that the affected tooth was Oehlers type IIIa. The root canals were expanded to a #90K file (Mani), and the invagination was expanded to a #50 HyFlex™ EDM finishing file (Coltene). To clean the root canals with complex morphology, circumferential filing was performed using an ultrasonic device, a #25 AM file (Satelec, Mérignac, France), a Sprason P-MAX (Satelec), and a #25 H file (Mani). The undercut area was cleaned using a pre-curved #25 AM file. The root canals were irrigated with 2.5% NaOCl and 3% EDTA and dried. A calcium hydroxide paste was placed on the root canals and the invagination, and a temporary blockade was performed. At the third visit (7 months after the start of treatment), the patient reported no clinical symptoms. Gutta-percha points (#90 for the root canals and #50 for the invagination) were used to confirm that the working length and cone fit were appropriate (Figure 3D), and the periapical lesion was healing. The root canals and invagination were irrigated using 2.5% NaOCl and 3% EDTA, with agitation using a NiTi instrument (XP-endo Finisher; FKG Dentaire, Neuchâtel, Switzerland) (Figure 3E) and passive ultrasonic irrigation (11). Following this, they were dried with paper points and filled using a single-cone hydraulic condensation technique (12) with a bioceramic root canal sealer (Bio-C Sealer; Angelus, Paraná, Brazil) (Figures 3F,G). After root canal filling, a periapical radiograph showed that the root canals and undercut were tightly filled with the sealer and gutta-percha (Figure 3H).

**Figure 4:**
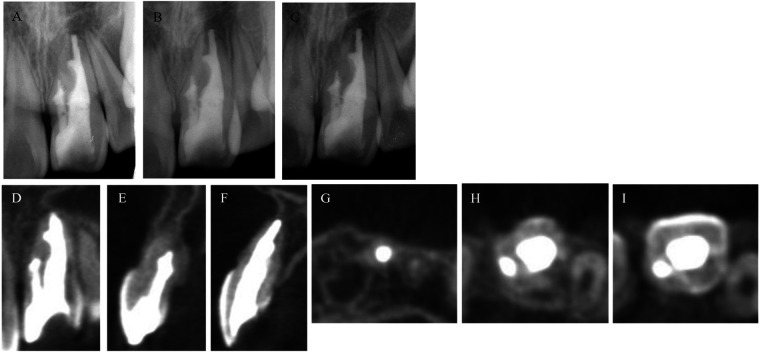
Post operative radiograph during 2 years follow up and CBCT images after 2 years follow-up. **(A–C)** Radiograph image of follow-up 6, 12 and 24 months. Two-year follow-up CBCT images of coronal cross section. The apical periodontal tissues were consistently stable and showed no tendency for recurrence. **(D–F)** Frontal and sagittal cross section CBCT images from the mesial to the distal regions, with no lesions observed at the root apex and a healed buccal cortical bone. **(G–I)** CBCT images of axial cross section from the apical to the coronal planes. No dissolution of the sealer was observed, and the root canal filling material was stable.

After confirming that there are no clinical symptoms 3 months after root canal filling, the access cavity was sealed with a bonding system (Clearfil Mega Bond FA; Kuraray, Chiyoda, Japan), a core resin (Clearfil DC Core Auto Mix One; Kuraray), and a composite resin (Clearfil Majesty ES Flow; Kuraray) (Figure 4A).

The patients were followed up at 6 months, 1 year, and 2 years after treatment (Figure 4). The clinical symptoms were also evaluated, and radiographs were taken (Figures 4A–D). A CBCT imaging for three-dimensional evaluation was scheduled at the 1-year follow-up visit but owing to the absence of symptoms and the patient's request, it was performed at the 2-year follow up. These images showed apical periodontal healing and stable root canal filling in the dens invaginatus (Figures 4E–J). At the 2-year follow-up after root canal filling, the lesion had healed (Figure 4E), and buccal cortical bone was observed on CBCT images (Figures 4F–J). No clinical symptoms or abnormal findings were observed 24 months after the nonsurgical root canal treatment. These treatments were performed by a resident with >2 years of experience in the endodontics department.

## 4 Discussion

In this case report, we present the nonsurgical root canal treatment of dens invaginatus in a maxillary central incisor in an 11-year-old girl whose preoperative dental radiographs suggested that the affected tooth was dens invagination. However, the presence of a connection between the invagination and the periapical tissue revealed that the tooth was Oehlers type IIIa. In type I, invagination occurs only at the crown and does not extend beyond the level of the external amelocemental junction and reported incidence rates of 69.8%–93.8% (13). In type II, the invagination extends to the root and does not communicate with the periodontal ligament, and, in some cases, the invagination and pulp cavities are connected. Reported incidence rates of 3.1%–26.6% (13). In type III, the invagination communicates with the periodontal ligament and does not connect to the pulp cavity and reported incidence rates of 3%–12.5% (13). Type IIIa communicates laterally with the periodontal ligament through a pseudo-foramen, and type IIIb communicates at the apical foramen.

The morphology of dens invagination varies in each patient, and no standardized treatment has been reported to date (3). Thus, it is important to develop a treatment plan according to the anatomy of the tooth, and precise imaging diagnosis is needed (3). Preoperative CBCT images are considered one of the factors that lead to successful nonsurgical root canal treatment (14). In this case, preoperative dental radiographs suggested that the affected tooth was dens invagination. However, the presence of a connection between the invagination and the periapical tissue revealed the tooth to be Oehlers Type IIIa. The treatment plan was developed using the preoperative CBCT images. The CBCT images revealed the absence of a connection between the invagination and the periodontal tissue; however, the invagination was connected to the periodontal tissue at the central portion of the root. CBCT imaging has certain limitations, and certain areas on the image cannot be read, such as narrowed root canals and apical foramen. Oehlers Types II and III are especially complex, and the connection between the invagination and the root canal or periodontal tissue should be checked during intraoperative visits. Additionally, dental operating microscopes are useful for exploring invaginations and preparing access cavities (3). For this patient, a microscope was used to search for the orifice of the invagination and to remove the infected dentin from the undercut (Figures 2A,B).

A continuous wave condensation technique using thermoplastic gutta-percha should be used for root canal filling of the dens invaginatus (12). Mineral trioxide aggregates and bioceramic materials with high biocompatibility are also useful (15), as are apexification (16) and regenerative endodontic therapies (17) for treatment of immature teeth. Preoperative CBCT evaluation revealed an open root apex. Some case reports have mentioned the effectiveness of the apical plug technique using hydraulic calcium silicate cement in cases of dens invaginatus with an open apex (18, 19). However, applying the apical plug technique to dens invaginatus requires advanced obturation skills. Air bubble during MTA filling may contribute to adverse effects. Although some clinical situations warrant the application of apical plug for the management of immature teeth, most immature teeth have a huge pulp space compared to matured teeth, making it easy to operate instruments in the space above the root canal. In this case, lateral condensation was also considered a clinical option; however, owing to the root canal morphology of a small orifice and pulp space with undercut, ensuring an appropriate taper was challenging, and there were concerns about the spreader gap and inability of adequate pressure application. Therefore, in our patient, the root canals were filled using a single-cone hydraulic condensation technique with a bio-ceramic sealer with high biocompatibility, sealing ability, and fluidity (3). Through injecting the sealer using an accessory tip and filling a root canal, an entire root canal and invagination, including the undercut, can be filled. The use of a highly fluid sealer in a root canal with uneven morphology allows the undercut to be filled (20). By ensuring the use of not only CBCT but also a dental operating microscope in combination with the complex shape of the dens invaginatus, we were able to accurately grasp its shape and address root canal treatment. Furthermore, even in root canals with complex shapes, including an open apex and undercuts, it was possible to perform root canal filling without dead space using the single-cone hydraulic condensation technique. This indicates that even in dens invaginatus with complex root canal morphology, it may be possible to switch from the traditional GP-centered root canal filling methods, such as lateral and vertical obturation, to a more sealer-based root canal filling. Owing to the structural complexity of dens invaginatus, a possibility of recurrence of apical periodontitis exists (21). Therefore, long-term follow-up is necessary in the future.

Although radiographic images revealed the open apex of the root, extrusion of the root canal sealer outside the apical foramen was not observed. This may be attributed to the influence of the calcium hydroxide dressing agent used. When the infection in the root canal is completely eliminated and a calcium hydroxide agent is applied, a mineralized barrier is formed at the root apex (22). In this case too, the application of a calcium hydroxide preparation may have induced the formation of calcified material at the root apex. A recent study reported that extrusion of root canal sealer outside the apical foramen does not affect spontaneous pain, healing, and clinical outcomes (23). In the future, through clinical research on malformed teeth, which can be effectively treated owing to advances in medical equipment, instruments, and materials, it will be necessary to discuss the recommended treatment methods based on the degree and condition of the malformed tooth. Thus, accumulation of similar cases and clinical studies are warranted. Notably, CBCT images taken two years after root canal filling showed that each undercut was tightly filled, with no observable lesion, while full healing was observed in the buccal cortical bone. Preoperative CBCT imaging of the root morphology, along with treatment under a microscope with a magnified view, appropriate mechanical cleaning, and chemical irrigation, helped reduce tooth infection. The use of a sealer with excellent flowability, sealing, and biocompatibility that could fill the undercut area also contributed to the favorable outcome for this patient.

The patient had poor oral hygiene since her first visit to our clinic, and plaque accumulation was noted on the palatal surface and in the invagination of the affected tooth, which presumably contributed to the infection in the invagination. The patient was given instructions on oral hygiene and dietary habits and efforts were made to control plaque, and improvements in oral hygiene were observed at the time of root canal filling. Plaque control through self-care in the invagination area is challenging; thus, regular cleaning and oral hygiene instructions are necessary.

Routine dental checkups may have resulted in earlier detection and treatment of the malformed tooth and preservation of the dental pulp.

A good outcome was achieved in a patient with a rare case of chronic apical periodontitis in a maxillary central incisor (Oehlers type IIIa) using CBCT imaging, dental operating microscopy, appropriate instrumentation, chemical cleaning, and a highly fluid sealer in the treatment plan.

## 5 Patient perspective

From the patient's perspective, the patient was able to receive treatment for a tooth that was considered difficult to treat. Although the length of treatment was longer because of the commute to school, the discomfort of the teeth disappeared, and the patient is now able to lead a normal daily life.

## Data Availability

The raw data supporting the conclusions of this article will be made available by the authors, without undue reservation.
